# Rapid and Quantitative Determination of S-Adenosyl-L-Methionine in the Fermentation Process by Surface-Enhanced Raman Scattering

**DOI:** 10.1155/2016/4910630

**Published:** 2016-10-13

**Authors:** Hairui Ren, Zhaoyang Chen, Xin Zhang, Yongmei Zhao, Zheng Wang, Zhenglong Wu, Haijun Xu

**Affiliations:** ^1^Beijing Bioprocess Key Laboratory, Beijing University of Chemical Technology, Beijing 100029, China; ^2^College of Science, Beijing University of Chemical Technology, Beijing 100029, China; ^3^Engineering Research Center for Semiconductor Integrated Technology, Institute of Semiconductors, Chinese Academy of Sciences, Beijing 100083, China; ^4^Analytical and Testing Center, Beijing Normal University, Beijing 100875, China

## Abstract

Concentrations of S-Adenosyl-L-Methionine (SAM) in aqueous solution and fermentation liquids were quantitatively determined by surface-enhanced Raman scattering (SERS) and verified by high-pressure liquid chromatography (HPLC). The Ag nanoparticle/silicon nanowire array substrate was fabricated and employed as an active SERS substrate to indirectly measure the SAM concentration. The linear relationship between the integrated intensity of peak centered at ~2920 cm^−1^ in SERS spectra and the SAM concentration was established, and the limit of detections of SAM concentrations was analyzed to be ~0.1 g/L. The concentration of SAM in real solution could be predicted by the linear relationship and verified by the HPLC detection method. The relative deviations (*δ*) of the predicted SAM concentration are less than 13% and the correlation coefficient is 0.9998. Rolling-Circle Filter was utilized to subtract fluorescence background and the optimal results were obtained when the radius of the analyzing circle is 650 cm^−1^.

## 1. Introduction

S-Adenosyl-L-Methionine (SAM) was discovered approximately 60 years ago. Since then, SAM has been identified with a wide spectrum of biological processes ranging from the synthesis of various neurotransmitters in the brain and catecholamine metabolism to gene expression and cell growth, differentiation, and apoptosis, to establish the “SAM empire” called by Cantoni [1–3]. Especially as a biological methyl group donor, SAM is used as a nutritional supplement and as a prescription drug for various diseases, including liver disease [4], osteoarthritis [5], neurologic syndrome [6], and depression [7]. Because SAM could only be ingested from the diet but could not be synthesized endogenously in the human body [4], the demands for SAM in synthetic medication and the nutritional supplements have received wild attention all over the world [8]. Therefore, an effective method for detecting SAM is beneficial to the investigation of the major role of SAM in cell function metabolism and the treatment of clinical disorders. Also the method can be used to optimize the fermentation conditions in fermentation process by monitoring the production of SAM. Recently, Sturgess measured SAM content of the commercially available supplements by high-pressure liquid chromatography-UV diode array detection and showed that the percentage of measured SAM was greater than threefold variation compared to the stated amount on the packaging [9]. Han et al. used high-pressure liquid chromatography (HPLC) to measure the production of SAM and L-isoleucine with 0.67 g/L and 13.8 g/L in* Corynebacterium* [10]. Hayakawa et al. reported that the regulation mechanisms are responsible for SAM production by ^13^C-metabolic flux analysis [11]. Notice that HPLC, as the major approach for accurate detection of SAM, is rather complex and takes time, implying that it is not suitable for real-time analysis in industry. This stimulates us to seek a novel method which can detect SAM concentration easily and quickly as well as cheaply.

Raman spectroscopy belonging to the molecular vibration spectrum permits the identification of numerous analytes with high discrimination because of its narrow spectral band response. However, the normal Raman signal is extremely weak because of its very small Raman scattering cross-section which limits seriously its applications in fast and sensitive detection. The Raman signals could be efficiently enhanced by surface-enhanced Raman scattering (SERS) technique when molecules were on or near the surface of certain noble metal structures with nanoscale features [12–14]. Silver nanoparticles had drawn particular attention for its potential as SERS substrate due to their highest electrical and thermal conductivities and strong surface plasmon resonance, which make them attractive for use in biochemistry. The enhancement factors (EFs) of SERS can be as much as 10^3^~10^7^ [15, 16]. Nowadays, SERS, which could minimize photobleaching, peak overlapping, and background signal in complex biological systems, was widely used as a signal-transduction mechanism in biological and chemical sensing [13]. Additionally, biological processes containing abundant materials need a more efficient and sensitive detection method [17]. So the SERS technique was applied to the detection of SAM. In this work, a three-dimensional and highly sensitive SERS-active substrate of highly ordered Si nanowire array (Si NWA) decorated by Ag NPs was prepared first, and the SERS spectra of SAM were investigated at various concentrations with the Ag/Si substrate (Ag/Si NWA). The limit of detections (LOD) of SAM was found experimentally to be as low as 0.1 g/L. Furthermore, according to the SERS signals of SAM with a concentration of 0.1~30 g/L, the linear relationship between the concentration of SAM and integrated intensity of peak centered at ~2920 cm^−1^ (S_2920_) was established, enabling the qualitative detection of SAM. As the practical application, the fast detection of SAM concentration can be considered as of two steps: S_2920_ was first measured in real solutions and then the SAM concentration was calculated based on the measured S_2920_ and the linear relationship. Meanwhile, the SAM concentrations could also be measured more accurately by HPLC for the verification of the calculation result. The relative deviations (*δ*) of the SAM concentration are defined as the ratio of the absolute value of the difference between SAM concentration with the fast detection method and that with HPLC method. The results confirm that *δ* are less than 13%.

## 2. Materials and Methods

SAM and the real solutions were obtained from the School of Life Sciences of Beijing University of Chemical Technology (SLS, BUCT). N-type (100) Si wafers (1~10 Ω·cm) purchased from the Tianjin Semiconductor Technology Research Institute were used as the substrate material. Acetone, alcohol, HF (≥40%), H_2_O_2_ (30%), silver nitrate, and deionized water were purchased from the Beijing Chemical Works. All chemical reagents were used as received without further purification.

Before deposition of Ag, Si wafer was first cut into dimensions of 1 × 3 cm^2^ and was then ultrasonically cleaned with acetone, ethanol, and distilled water for 5 min, respectively. Next the clean Si wafer was dried in air. The wafer was placed in a mixture of 4.6 M HF and 0.005 M AgNO_3_ for 1 min and then washed with deionized water several times to remove excess Ag particles. Ag nanoparticles (NPs) were then deposited on the surface of the Si wafer by placing the wafer in a mixture 4.6 M HF and 0.5 M H_2_O_2_ at room temperature for 60 min. After that the Si wafer was taken out steadily. Finally, it was kept in 1 M AgNO_3_ solution for 1 min and dried in air.

The concentration of SAM of real fermentation system is from 1 g/L to 14 g/L [18]. Based on the master facility, the solutions of SAM were prepared with concentration ranges of 0.01~30 g/L by diluting the stock solution of SAM (30 g/L) with the minimum concentration which could be tested by SERS. The concentration range is enough to meet the demand for the detection of SAM in real fermentation process. SERS detection of SAM had been completed in 2 hours to ensure the bioactivity. The fermentation liquids were provided by SLS and BUCT with no operation. The cells were disrupted by sonication for 10 min on ice and then separated by centrifugation at 1000 rpm for 10 min at 0°C from the fermentation liquids. The SAM in supernatant was tested by SERS.

SERS measurements were performed at room temperature on a LabRAM ARAMIS Raman system with the 632.8 nm laser as excitation. The diameter of the light spot area was ~1 *μ*m and the incident power was 9 mW. The spectral resolution was 1 cm^−1^ and the spectra were recorded with an accumulation time of 15 s. The SERS substrates were preimmersed in corresponding solution for 30 min to ensure adsorption equilibrium, then rinsed with deionized water, and dried in a vacuum chamber for 5 min at 25°C before each SERS measurement. The range of SERS detection through the whole process was from 550 to 3100 cm^−1^ at the same condition. The Raman band of a Si wafer at 520.7 cm^−1^ was used to calibrate the spectrometer.

The background with no chemical information caused by the fluorescence effect in obtained Raman spectrum should be removed. The Rolling-Circle Filter (RCF) is an intuitive filter algorithm to subtract background effect. There are two main advantages of the RCF: (1) it has a single parameter (radius) and (2) there is no restriction on the background shape. This method is based on the geometrical difference between characteristic peaks and background [19–21]. The algorithm and a code written in Matlab language are given in Supplementary Information (see Supplementary Material available online at http://dx.doi.org/10.1155/2016/4910630).

## 3. Results and Discussion

**Figure 1:**
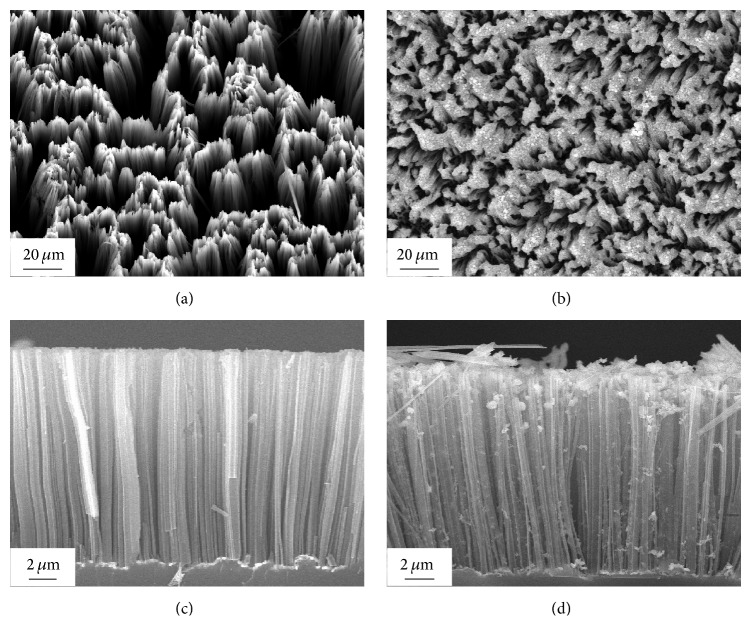
FE-SEM images of a top view and cross-sectional side view of the SiNWA (a), (c); top view and cross-sectional side view of the Ag/SiNWA (b), (d).

We select highly ordered porous Si NWA as a template to fabricate Ag-based nanostructured SERS-active substrates where large-quantity Ag NPs with appropriate and uniform size were assembled on the surface of Si NWA via the reaction between Ag ions and Si-H bonds. Compared with SERS substrates based on Ag nanoparticle films on monocrystalline Si substrate, the porous Si NWAs have much wider specific areas, allowing an increased loading of Ag NPs and adsorption of target molecules, as well as the higher possibility of forming “hot spots.” The typical morphologies of Si NWA and Ag/Si NWA were shown in Figure 1. From the top (Figure 1(a)) and cross-sectional (Figure 1(c)) views of Si NWA, Si NWs are distributed uniformly on the whole wafers and are oriented perpendicularly to the substrate surface with good uniformity. The diameters of Si NWs are in the range of 300~1000 nm, while the length is around 10 *μ*m. From the amplified FE-SEM image of the top view of Si NWA, as shown in the inset of Figure 1(a), it can be observed that the upper ends of Si NWs bend towards each other to form small bunches. The porous nanostructures of Si NWs provide a much larger surface area, which is of great significance to load Ag NPs and to enhance light trapping. After the Si NWA is decorated, the representative top (Figure 1(b)) and cross-sectional (Figure 1(d)) FE-SEM images of Ag/Si NWA, which also intuitively show that substantive Ag NPs are attached to the surface of each Si NW, and a complex and unique nanocomposite array were formed. The Raman enhancement effect of the Ag/Si NWA substrate is studied, and an EF as large as 3.33 × 10^5^ is calculated, as shown in Supplementary Figure S1. The high EF value indicates Ag/Si NWA has a much better Raman enhancement effect than Si NWA, which is favorable to ultrasensitive detection.

**Figure 2:**
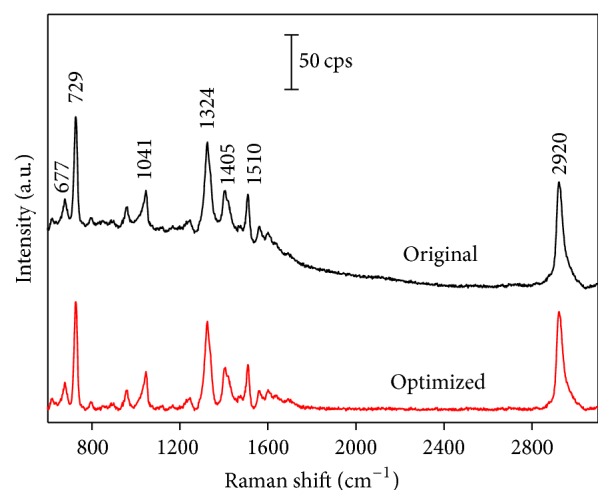
Model spectrum (black curve: 10 g/L) and the results of applying the RCF method to the curves (red curve: parameters of the analyzing circle *R* = 650 cm^−1^ and *R*_*x*_/*R*_*y*_ = 16 cm^−1^/rel. unit).

Figure 2 shows the original spectrum (black curve) and the optimized spectrum (red curve) at 10 g/L of SAM concentration. It could be found that the fluorescence background with no chemical information was removed by RCF processing. There is no obvious visible distortion in the optimized curve either. The prominent Raman peaks of SAM at about 677, 729, 1041, 1324, 1405, 1510, and 2920 cm^−1^ were assigned to *ν* (CS), *ν*_*s*_ (CN), *ω* (CH2), *ν* (CC), *ν*_as_ (CN), and *ν* (CH_3_), respectively [22, 23]. The radius of RCF, as a key parameter, was analyzed to achieve the optimal results. Supplementary Figure S2(a) shows the original spectra and the results after applying the RCF with various radii (50, 200, 400, 650, 800, 1000, 1200, and 1600 cm^−1^). Supplementary Figure S2(b) shows how the difference value between the spectra optimized by the RCF and that with the background subtracted manually varies with the radius of RCF. From Supplementary Figure S2(b), it could be concluded that the optimal radius *R* is around 650 cm^−1^. When *R* > 650 cm^−1^, the background is partly subtracted, and when *R* < 650 cm^−1^, the Raman spectra may have distortion. Therefore, it is verified that *R* of RCF is significantly greater than the Raman line width and less than the radius of curvature of the background in the spectrum [24].

**Figure 3:**
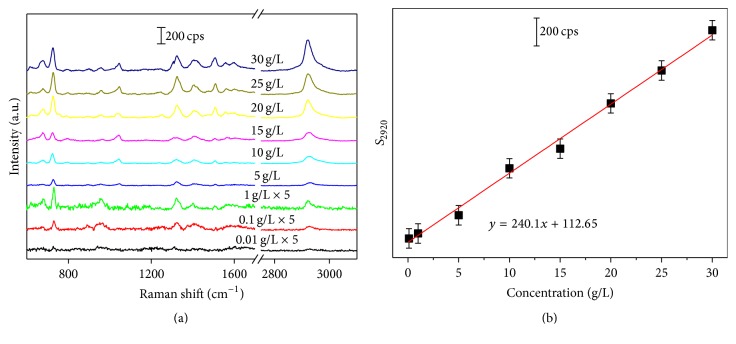
The SERS spectra of SAM solution in various concentrations (a), the quantitative relation curve of the peak intensities centered at ~2920 cm^−1^ of SAM (b). Each sample was measured for 5 times and the average was calculated.

**Figure 4:**
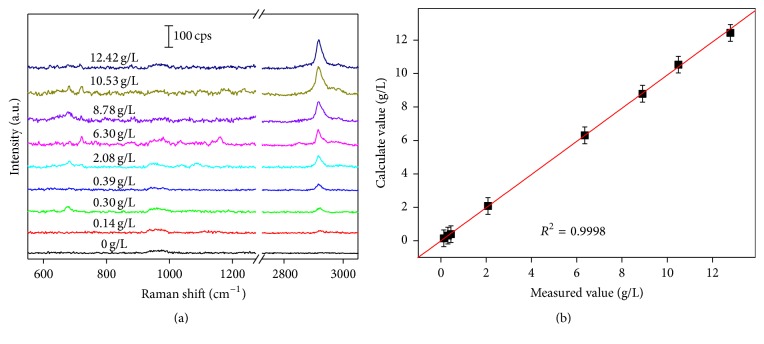
SERS spectra of SAM solution in different concentration (a). Plot for the comparison of the measured value with the calculated value (b). Each sample was measured for 5 times and the average was calculated. The SAM solution of 0 g/L was used to prove that the peak centered at ~2920 cm^−1^ was produced by SAM.

Usually, the statistical approaches such as partial least square regression (PLSR), Support Vector Machine (SVM) [25], and Gaussian Mixture Discriminant Analysis [26] are combined with principal components analysis (PCA) [27] to quantitatively analyze mixture by Raman or SERS. In the SAM fermentation solution, there is a mass of materials, including the water, inorganic salt, alcohol, ketone with no obvious Raman signals, and SAM with sharp characteristic peaks. With single analyte, we introduce the linear analysis with the relationship between Raman intensity and the concentration of SAM. Figures 3(a) and 3(b) show, respectively, the detection of 0.01 to 30 g/L SAM and the establishment of linear relationship between SAM concentration and S_2920_. As shown in Figure 3(a), the SERS spectra exhibit good regularity, and the intensity recedes with the decrease of SAM concentration. Based on the data presented in Figure 3(a), the relationship curve between the S_2920_ (average and standard deviation from 8 samples) and the concentration was established. Clearly, the dependence of S_2920_ on the SAM concentration is nearly linear over the concentration range which was depicted in Figure 3(b). This linear relationship (*y* = 240.10*x* + 112.65) between S_2920_ and SAM concentration allows for the calibration of our substrate to determine unknown concentration of SAM in solutions. As shown in Supplementary Figure S3, there is no obvious Raman spectrum when the SAM concentration decreases to 0.01 g/L whether on Ag/Si NWA or Si NWA substrate, and SERS signals can be merely detected at the concentration of 0.1 g/L. Thus, LOD of SAM was determined to be 0.1 g/L, and the linear relationship established above is applicable in the range from 0.1 g/L to 30 g/L, allowing for the quantitative determination of SAM. In order to investigate whether the concentration of SAM could be assessed by SERS technology in real biological samples, we try to study the application of Raman spectrum in real solutions containing SAM at different concentration. The corresponding Raman spectra could be seen in Figure 4. As shown in Figure 4(a), the spectra showed obviously that Raman peaks were centered at 970 cm^−1^ and 2920 cm^−1^. The SERS intensity of the mineral salts and glucose contained in real solutions was so weak that it could not be obviously observed. The Raman peaks centered at 970 cm^−1^ and 2920 cm^−1^ were assigned to Si NWA and the CH_3_ symmetric stretching of SAM, respectively. The area of peak centered at 2920 cm^−1^ showed an increasing trend with concentration of SAM which verified that the Raman peak at 2920 cm^−1^ was aroused by SAM. Based on the linear relationship in Figure 4(b), the SAM concentration could be calculated and the results were listed in Table 1.

HPLC analysis of real solutions had also been applied to verify the linear function relation and the corresponding results were also listed in Table 1. The measured value and error of the real solutions were presented, which validates the consistency and the accuracy of the linear relationship. Comparing these data, *δ* less than 13% (including two bigger relative deviations with 12.6% and 11.13% and others less than 5%) were obtained. To present the verification results more intuitively, the predicted concentrations of SAM as a function of the value obtained by HPLC were plotted and shown in Figure 3(b). The correlation coefficient square (*R* ^2^) is 0.9998. Therefore, the determination data were well consistent with the calculated data, indicating that a new method of quantitative determination of concentration of SAM has been developed based on the Ag/Si NWA SERS substrate.

## 4. Conclusions

A new method, surface-enhanced Raman scattering, to detect the SAM concentration rapidly and conveniently was proposed by us. In the investigation carried out here, the linear relationship (*y* = 240.10*x* + 112.65) between the concentration of SAM and the integrated intensity of peak centered at ~2920 cm^−1^ was established. Experimentally the LOD of SAM is 0.1 g/L. As the concentration of SAM is increased, the integrated intensity of peak increases accordingly. The linear relationship has the same dependence of SAM concentration in aqueous solutions and the real solutions, due to the weak influence on 2920 cm^−1^ of other contents in biological cells. The relative deviations are less than 13%, and the correlation coefficient is 0.9998. This investigation provided a possibility for carrying out rapid detection on SAM. With the mathematic approaches used to establish the predictable relationship including RCF and linear analysis, this research provided a reference to study the relationship between the concentration of SAM and the SERS signals. Furthermore, the results obtained may be applied to the detection for synthesis of SAM and the SAM contention in biological process.

## Supplementary Material

The algorithm of Rolling-circle spectral filter was presented to reduce the backgrounds. Eight radius (R) values were proposed to optimize Raman spectra, and 650cm^−1^ was selected as the optimized R with the background subtracted effectively. In order to understand the ability for the Ag/Si nanowire array to enhance the Raman signal of SAM, we calculated the enhancement factor with high value of 3.33×10^5^ which indicates Ag/Si NWA had a better Raman effect. Meanwhile the limit of detection was determined to be 0.1 g/L with no obvious characteristic peaks of SAM samples of 0.01g/L.

## Figures and Tables

**Table 1:** The calculated and measured results of 8 samples with the relative deviations.

Sample (SAM)	S_2920_	Calculated values	HPLC measured values	*δ* (%)
1	147.30	0.14	0.13	12.60
2	185.76	0.30	0.31	0.29
3	206.76	0.39	0.44	11.13
4	611.42	2.08	2.08	0.13
5	1625.38	6.30	6.36	0.87
6	2221.81	8.78	8.91	1.36
7	2640.04	10.53	10.49	0.31
8	3095.71	12.42	12.79	2.89
