# Supplementary material for: Rapid and Quantitative Determination of S-Adenosyl-L-Methionine in the Fermentation Process by Surface-Enhanced Raman Scattering
Source: J Anal Methods Chem. 2016 Oct 13;2016:4910630. doi: 10.1155/2016/4910630 (PMC5081456; doi:10.1155/2016/4910630)
Supplement: Supplementary file 1 — The algorithm of Rolling-circle spectral filter was presented to reduce the backgrounds. Eight radius (R) values were proposed to optimize Raman spectra, and 650cm−1 was selected as the optimized R with the background subtracted effectively. In order to understand the ability for the Ag/Si nanowire array to enhance the Raman signal of SAM, we calculated the enhancement factor with high value of 3.33×10^5^ which indicates Ag/Si NWA had a better Raman effect. Meanwhile the limit of detection was determined to be 0.1 g/L with no obvious characteristic peaks of SAM samples of 0.01g/L. [file 4910630.f1.pdf]

Supplementary information

Rapid and Quantitative Determination of S-Adenosyl-L-Methionine in the Fermentation Process by Surface-enhanced Raman Scattering

Hairui Ren,[1,2] Zhaoyang Chen,[1,2] Xin Zhang,[1,2] Yongmei Zhao,[3] Zheng Wang,[1,*] Zhenglong Wu,[4] and Haijun Xu[1,2,*]

[1]*Beijing Bioprocess Key Laboratory, Beijing University of Chemical Technology, Beijing, 100029, PR China*

[2]*College of Science, Beijing University of Chemical Technology, Beijing, 100029, PR China*

[3]*Engineering Research Center for Semiconductor Integrated Technology, Institute of Semiconductors, Chinese Academy of Sciences, Beijing, 100083, PR China*

[4]*Analytical and Testing Center, Beijing Normal University, Beijing, 100875, PR China*

*Authors to whom correspondence should be addressed; electronic mails: wangzhengbio@163.com (Z. Wang), hxju@mail.buct.edu.cn (H. Xu)

RCF algorithm: A rolling-circle spectral filter (RCF) method proposed by Mikhailyuk et al was utilized to remove the background of Raman spectra. The principle of this method is that the curvature radii of the Raman peaks and the backgrounds are different. Therefore, a certain radius R which is significantly greater than the Raman linewidth and less than the curvature radius of the background should be determined. Eight radius values were used as the candidates and 650 cm^{-1} was selected as the optimized R. The input array contains the coordinates of points of the original spectrum. In the beginning, the output array is identical to the input array. A circle is constructed for each point of the input array starting from the first point. The abscissa of the center of the circle coincides with the abscissa of the corresponding point. The spectrum and the circle have at least one common point, while the ordinates of the remaining points of the circle are less than the ordinates in the input array. Thus, the circle lies below the spectrum. The differences between the ordinates in the input array and the ordinates of the upper arc of the circle are compared to the corresponding ordinates of the output array. The minimum of two values becomes the new ordinate of the output array. Then, the procedure is repeated for the next point, so that the output array is modified many times. Thus, a circle rolls under the spectrum and subtracts the fragments of the curve whose radius of curvature is greater than R. At a certain radius of the circle, the background is effectively subtracted, whereas the Raman lines remain virtually unchanged. The key advantage of RCF is that we only use a single parameter (radius) in the RCF process. Besides, we should normalize the spectrum before applying the RCF in next step.

$$X\ norm[i] = (X[i] - \min[X] / \max[X] - \min[X]) \cdot N$$

Where X is the spectrum intensity, N is the number of all spectral points. MATLAB 2014 is used to implement and build the algorithm of RCF.

EF algorithm: In order to understand the ability for the Ag/Si nanowire array (Ag/Si NWA) to enhance the Raman signal of SAM, several tests are carried out. Because the Raman signals of the SAM solutions were not observed distinct, the R6G was carried out to detecte the Raman signals. To determine the enhancement factor (EF) of the Ag/Si NWA composite structure, a Si NWA without Ag NPs is used as a reference along with the following standard equation:

$$\mathbf{EF} = \frac{I_{SERS}}{I_{Raman}} \times \frac{N_{Raman}}{N_{SERS}} \times \frac{P_{Raman}}{P_{SERS}} \times \frac{T_{Raman}}{T_{SERS}} = \frac{I_{SERS}}{I_{Raman}} \times \frac{N_{Raman}}{N_{SERS}}$$

Where the parameters P and T represented the laser power and the acquisition time, respectively. In the measurement, we carefully kept the same experimental conditions (laser power 9 mW, acquisition time 15 s, laser spot size 1 μm in diameter) to make the ratios of P_{Raman}/P_{SERS} and T_{Raman}/T_{SERS} equal 1. I_{SERS} and I_{Raman} are the integrated Raman intensities of a specific SERS peak observed for the solutions adsorbed on Ag/Si substrate and Si substrate, respectively; N_{SERS} and N_{Raman} are the numbers of molecules adsorbed on the surface of the corresponding substrates. Here the Raman band centered at 773 cm^{-1} was chosen to calculate I values. The conventional Raman spectrum was collected when a 10 μl 10^{-3} M R6G loaded on a 1×1cm^2 Si substrate and the integrated peak intensity I_{Raman} was measured to be ~3004.75. N_{Raman} was determined based on the 10^{-3} M R6G solution and the illuminated volume (V_{illu}) of our Raman system. For our Raman setup, the illumination focus has a diameter of the laser spot size ~1 μm and the penetration depth of 633 nm laser beam is ~3mm. As a result, V_{illu} is 2.36×10^3 μm^3 and N_{Raman} is 1.42×10^9. When determining N_{SERS} in the illuminated volume of our Raman setup, we assumed that R6G molecules were absorbed as a monolayer on the surface of Ag/Si substrate. The surface area of one R6G molecule is ~2.0 nm^2, which was calculated from the geometric area of length (1.37 nm) × width (1.43 nm) of one R6G molecule. As show in figure X, the corresponding integrated peak intensity (I_{SERS}) of the 773cm^{-1} Raman band was significantly enhanced with increasing R6G concentration from 10^{-5} M to 10^{-3} M, but the ISERS values of 10^{-2} M and 10^{-1} M are very close to that of 10^{-3} M. Consequently, the surface of the Ag/Si substrate is presumed to be fully surrounded with R6G molecule when its concentration reaches 10^{-3} M, thus yielding N_{SERS} is 3.93×10^5 and the corresponding I_{SERS} is 246360.75. The ratios of N_{Raman}/N_{SERS} and I_{SERS}/I_{Raman} were estimated to be 3.61×10^3 ($1.42\times10^9/3.93\times10^5$) and ~92.25 (246360.75/3004.75). Accordingly, the proposed SERS-active substrate of the Ag/Si exhibits a large EF of 3.33×10^5, which is sufficient the ultrasensitive detection. The same EF magnitude was obtained for the other fingerprint Raman peaks. The high EF value indicates Ag/Si NWA had a better Raman enhancement effect.

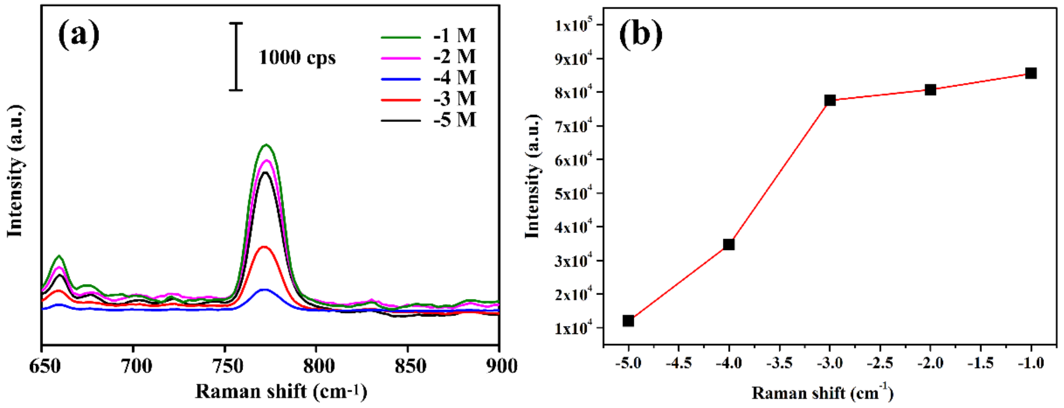

Fig. S1 SERS spectra of R6G molecule collected on the random-selected 10 places of the Ag/Si substrate with different concentrations **(a)**, the integrated intensities of the peak centered at 773 cm^{-1} versus R6G concentration **(b)**.

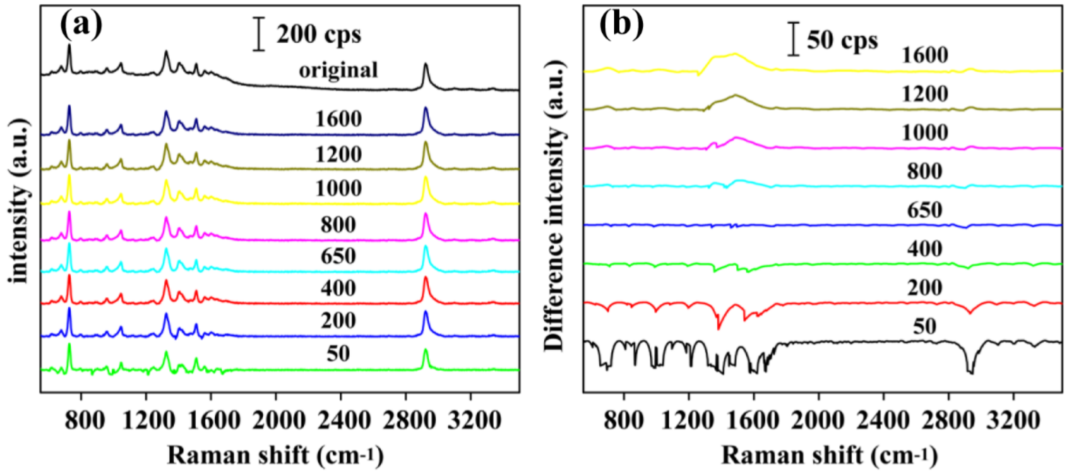

Fig. S2 The results of RCF processing at different radius **(a)**. The different values between the results of RCF processing and the spectra with the background subtracted manually **(b)**.

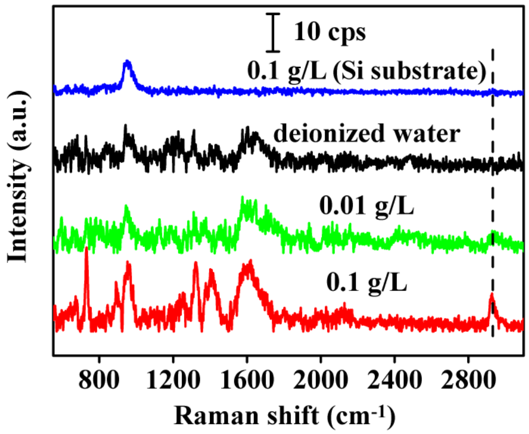

Fig. S3 SERS spectra of deionized water and two SAM samples (0.1 g l^{-1} and 0.01 g l^{-1}), and the Raman spectra of SAM samples of 0.1 g 1^{-1}.
